# Comparative transcriptome analysis of geographically distinct virulent and attenuated *Babesia bovis* strains reveals similar gene expression changes through attenuation

**DOI:** 10.1186/1471-2164-14-763

**Published:** 2013-11-06

**Authors:** Monica J Pedroni, Kerry S Sondgeroth, Gina M Gallego-Lopez, Ignacio Echaide, Audrey OT Lau

**Affiliations:** 1 Program of Genomics, Department of Veterinary Microbiology & Pathology, College of Veterinary Medicine, Washington State University, ADBF 4043, Pullman, WA, , 99164, USA; 2 Paul G. Allen School for Global Animal Health, College of Veterinary Medicine, Washington State University, Pullman, WA 99164, USA; 3 Laboratorio de Inmunología y Parasitología, Instituto Nacional de Tecnologia Agropecuaria (INTA), Rafaela CP2300, Argentina

**Keywords:** *Babesia bovis*, Apicomplexans, Virulence, Attenuation, Transcriptome, Microarray, RNA-sequencing

## Abstract

**Background:**

Loss of virulence is a phenotypic adaptation commonly seen in prokaryotic and eukaryotic pathogens. This mechanism is not well studied, especially in organisms with multiple host and life cycle stages such as *Babesia,* a tick-transmitted hemoparasite of humans and animals. *B. bovis,* which infects cattle, has naturally occurring virulent strains that can be reliably attenuated *in vivo*. Previous studies suggest the virulence loss mechanism may involve post-genomic modification. We investigated the transcriptome profiles of two geographically distinct *B. bovis* virulent and attenuated strain pairs to better understand virulence loss and to gain insight into pathogen adaptation strategies.

**Results:**

Expression microarray and RNA-sequencing approaches were employed to compare transcriptome profiles of two *B. bovis* strain pairs, with each pair consisting of a virulent parental and its attenuated derivative strain. Differentially regulated transcripts were identified within each strain pair. These included genes encoding for VESA1, SmORFs, undefined membrane and hypothetical proteins. The majority of individual specific gene transcripts differentially regulated within a strain were not shared between the two strains. There was a disproportionately greater number of *ves* genes upregulated in the virulent parental strains. When compared with their attenuated derivatives, divergently oriented *ves* genes were included among the upregulated *ves* genes in the virulent strains, while none of the upregulated *ves* genes in the attenuated derivatives were oriented head to head. One gene family whose specific members were consistently and significantly upregulated in expression in both attenuated strains was spherical body protein (SBP) 2 encoding gene where SBP2 truncated copies 7, 9 and 11 transcripts were all upregulated.

**Conclusions:**

We conclude that *ves* heterodimer pair upregulation and overall higher frequency of *ves* gene expressions in the virulent strains is consistent with the involvement of this gene family in virulence. This is logical given the role of VESA1 proteins in cytoadherence of infected cells to endothelial cells. However, upregulation of some *ves* genes in the attenuated derivatives suggests that the consequence of upregulation is gene-specific. Furthermore, upregulation of the spherical body protein 2 gene family may play a role in the attenuated phenotype. Exactly how these two gene families may contribute to the loss or gain of virulence is discussed.

## Background

During pathogenesis, it is advantageous for a pathogen to adjust to the shifting selective pressures exerted by the host. Depending on the environmental milieu there may be strong selection for specific pathogenic phenotypes such as virulence or attenuation acquisition. These phenotypic shifts may be achieved through sexual reproduction where novel recombinations often lead to genome diversity [1]. However for many multi-stage pathogens, this cannot occur during haploid life stages and thus, they depend on genomic mutations or adaptations through non-mutational mechanisms such as phenotypic variation. Mutations associated with virulence (or attenuation) are frequently documented in eubacteria [2-4] and viruses [5,6]. However, there is a significant gap in knowledge of virulence-associated gene mutations in protozoa, including pathogens that have major impact on global public and animal health. With increased emphasis on the development and delivery of vaccines for hemoparasites such as *Babesia*, *Theileria*, and *Plasmodium* spp. [7-10], the ability to predictably and stably attenuate pathogens would be a significant step toward disease control strategies.

*Babesia bovis* is a tick-borne apicomplexan protozoan responsible for causing bovine babesiosis [11]. Virulent strains are capable of inducing cerebral babesiosis where >90% of erythrocytes sequestered in cerebral capillaries are infected with *B. bovis* parasites. The resulting neurovirulent phenotype is clinically similar to cerebral malaria caused by *P. falciparum*[12]. In nature, there is a wide diversity of *B. bovis* virulent phenotypes [13] but experimentally, attenuation can be predictably induced *in vivo* by serial passages of virulent *B. bovis* in a splenectomized host. The phenotypic characteristic of neurovirulence is gradually lost, and an attenuated derivative is obtained. Animals infected with the attenuated derivative are protected upon virulent parental challenge, indicating that determinants of virulence may be modulated independently of epitopes responsible for protective immunity. The absence of the spleen during the attenuation process represents a change in the host environment and subsequent change in selection pressure. Although the mechanism of attenuation is undetermined, one plausible explanation is that circulating parasites are not cleared as they would be in spleen-intact animals, resulting in the sequential enrichment of a non-virulent parasite subpopulation.

Recent genomic comparison between three virulent and attenuated *B. bovis* strain pairs indicate that there are no consistent changes among the protein-coding genes shared between strain pairs that could explain the divergent phenotypes. However, an overall genome reduction in all three attenuated strains was observed [14]. These data suggest that (i) differences between parental and derivative strain pairs may lie in the non-coding regions which influence transcriptomic variability between virulent and attenuated strains, (ii) the loss of genome content during the attenuation process is the key to attenuation, or (iii) both.

In this study, we investigated the transcriptome profiles of two geographically unrelated *B. bovis* strain pairs to determine if attenuation is a result of common or distinct strain-specific transcriptomic regulation. Although both *B. bovis* strains were subjected to identical treatment/selection pressure for attenuation acquisition, transcriptome profiles of these two strains through attenuation were different. Detailed discussion of differential gene expression in their contribution to attenuation is discussed.

## Results and discussion

### T2Bo and L17 transcriptome approaches

In order to determine if virulence loss is controlled at the transcriptional level, we compared the global transcriptomes of two strain pairs of *B. bovis*. These strain pairs, T2Bo_*V*irulent parent and *A*ttenuated derivative and L17_*V*irulent parent and *A*ttenuated derivative, are geographically distinct field isolates from Mexico and Argentina, respectively [14]. Biological replicate (BR) sample pairs were prepared for transcriptomic analyses. For the generation of BR samples, animals were inoculated with 1 × 10^7^ parasitized erythrocytes. When clinical symptoms associated with acute babesiosis were observed and blood smears detected parasitemia, infected blood was collected to establish short-term (<30 days) culture to obtain sufficient starting material for RNA extraction (TriZol and RNeasy Mini-elute kit, Invitrogen. This short-term *in vitro* culture of virulent and attenuated *B. bovis* followed by *in vivo* evaluation of phenotype in infected animals were performed to ensure the original phenotypes were maintained post cultivation (unpublished data). Using the published T2Bo genome (http://www.piroplasmaDB.org), we generated an expression microarray that represented >99.4% of the protein-coding genes to investigate transcriptome differences between T2Bo_V and _A. Due to the incomplete L17 genome, L17_V and _A transcriptome comparison was performed by RNA-sequencing. The utilization of the T2Bo genome-based microarray for L17 transcriptome investigation would reflect gene expressions shared between T2Bo and L17 and exclude L17-specific transcripts as gene family member variations are known to exist between different *B. bovis* strains [15].

### Expression microarray analysis of *B. bovis* T2Bo transcriptomes

**Figure 1:**
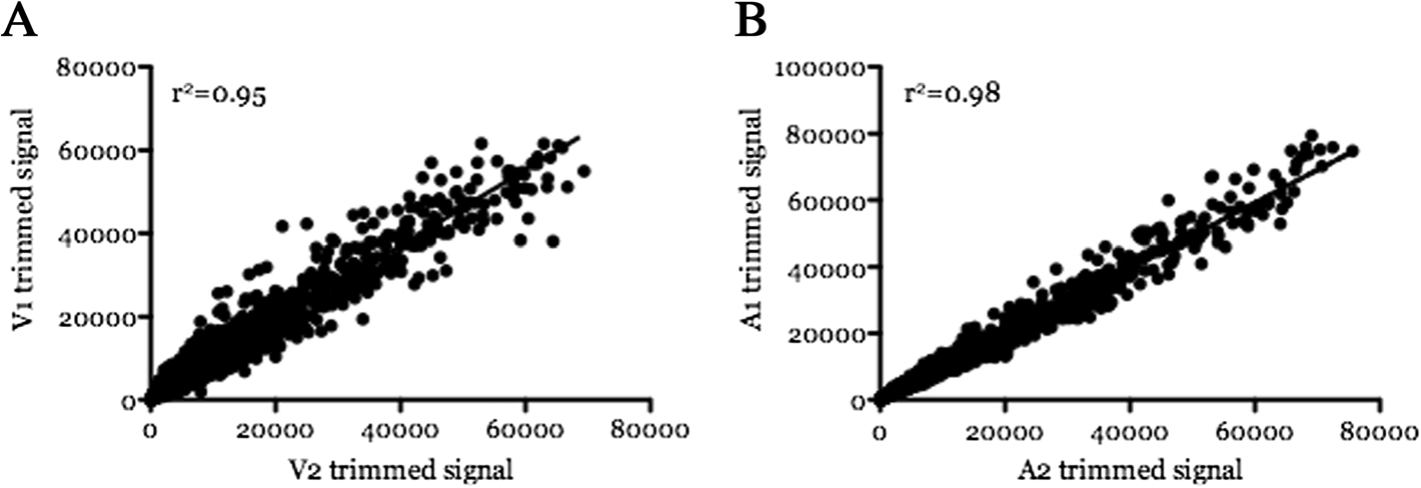
**Correlation plots of trimmed signal intensities between the two T2Bo biological replicate (BR) sample pairs.** Signal intensities are the sum of the pixel signals in the area of interest minus the scaled 2-pixel median background. The intensities are then median-normalized by calculating the median of all spots. Comparisons of each virulent pair **(A)** and each attenuated pair **(B)** are shown.

Two independently produced biological replicates (BR) were used to prepare for the virulent and attenuated samples in the microarray analysis while a third BR sample pair was used for assay validation. The microarray data reveal the total number of detectable transcripts ranged from 78 to 89% of all predicted transcripts depending on the BR samples (Table 1). Both biological replicate sample pairs were highly correlated with coefficient of determination (R^2^) trimmed signal values between both BR sample pairs to be 0.95 and 0.98 (Figure 1A and B).

**Table 1:** Summary of the T2Bo genome-based microarray statistics

	**T2Bo_A1**	**T2Bo_V1**	**T2Bo_A2**	**T2Bo_V2**
# genes detected	3,210	3,293	2,896	3,004
% genes detected	86.8	89.0	78.3	81.2
	**T2Bo_A1 vs. V1**		**T2Bo_A2 vs. V2**	
# differentially expressed genes*	749		773	
# differentially expressed genes with 2x difference	89		64	

*Student *T*-test, p < .01; DE, differentially expressed.

**Figure 2:**
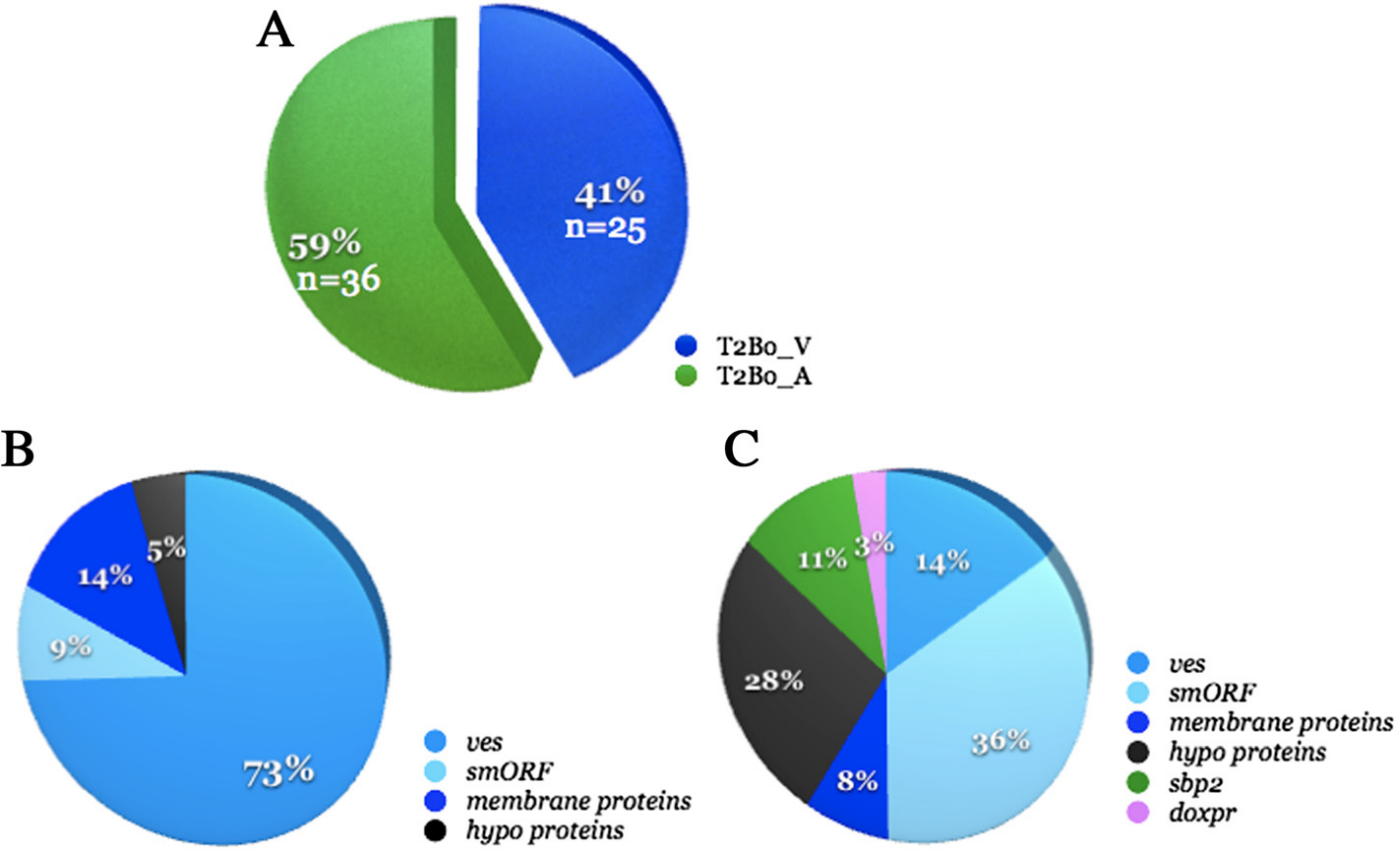
**Global distribution of differentially regulated transcripts in virulent and attenuated T2Bo *Babesia bovis *strain pair and the breakdown of the putative functions of these transcripts. (A)** Sixty-one genes were differentially expressed where 41% and 59% were upregulated in the virulent and attenuated strain, respectively. **(B)** Significantly upregulated genes in the virulent strain with corresponding putative functions. **(C)** Significantly upregulated genes in the attenuated derivative strain and their predicted functions. *Ves,* gene encoding for the variant erythrocyte surface antigen (VESA)1; *sbp2,* gene encoding for spherical body protein 2; *smORF,* gene encoding for small open reading frame protein; *doxpr,* gene encoding for 1-deoxy-D-xylose-5-phosphate reductoisomerase.

Microarray-based transcriptome analysis indicated that 61 genes in BR1 and 2 were differentially regulated at significant levels (>2 fold difference, p < .05) between V and A strain pairs (Figure 2A). This equates to 1.66% of the total detectable transcripts within the genome. Among them, 25 and 36 were upregulated in the virulent (T2Bo_V) parent and its attenuated (T2Bo_A) derivative, respectively (Figure 2A, Additional file 1: Table S4-5). Seventy three percent of the transcripts upregulated in T2Bo_V were variant erythrocyte surface antigen (*ves*) genes followed by genes encoding for unspecified putative membrane proteins at 14% (Figure 2B). *Ves* is the largest gene family in *B. bovis* and encodes the *v*ariant *e*rythrocyte *s*urface *a*ntigen (VESA)-1 in which divergently oriented *ves* α and β are hypothesized to be transcribed at the locus of active transcription, resulting in the expression of a VESA1 αβ heterodimer on the erythrocyte surface [16]. Microarray data reveal that seven and eight *ves* α and β genes, respectively, were upregulated in T2Bo_V and among them, three α and β are paired in divergently oriented manner as described previously [16]. Al-Khedery and Allred showed that divergently oriented *ves* α and β are transcriptionally regulated coordinately and that they function in antigenic variation and cytoadhesion. *Ves* genes that are not oriented in the same manner may act as donor sequences in segmental conversion. Interestingly, although there were upregulated *ves* α and β genes in T2Bo_A as well, none of them (nα = 2 and nβ = 3) are paired in divergently oriented manner on the chromosomes. Based on this finding, it is plausible to hypothesize that (i) transcription of *ves* α and β divergently oriented pair might be impaired in T2Bo_A or (ii) that parasites expressing divergently oriented *ves* α and β may not be numerous enough for their transcripts to be detected by the array approach in the samples. Nonetheless, either scenario may contribute to the observed lack of neurovirulence in animals in animals infected with T2Bo_A.

In addition to *ves* genes that were upregulated in both parental virulent and attenuated T2Bo, two (9%) *smORF* (*sm*all *o*pen *r*eading *f*rame) genes were upregulated in T2Bo_V while 13 *smORF* (36%) were upregulated significantly in T2Bo_A (Figure 2B-C). *SmORF* is the second largest gene family with 43 members in T2Bo [17]. Although the function of the SmORF proteins is unknown, the proximity of these genes with *ves* led to speculation that their expression may be coordinated with *ves’*[18]*.* However, since only two *smORF*s but 16 *ves* genes in T2Bo_V, and 13 *smORF*s but five *ves* genes in T2Bo_A, were upregulated, and none of the *smORF* gene locations were associated with those of *ves, smORF* gene transcriptional regulation may be independent from *ves* after all. The higher frequency of upregulated *smORF* gene transcripts in T2Bo_A is noteworthy*,* but its transcription in *B. bovis* is likely to be independent of the virulence/attenuation phenotype (Figure 2B-C).

**Figure 3:**
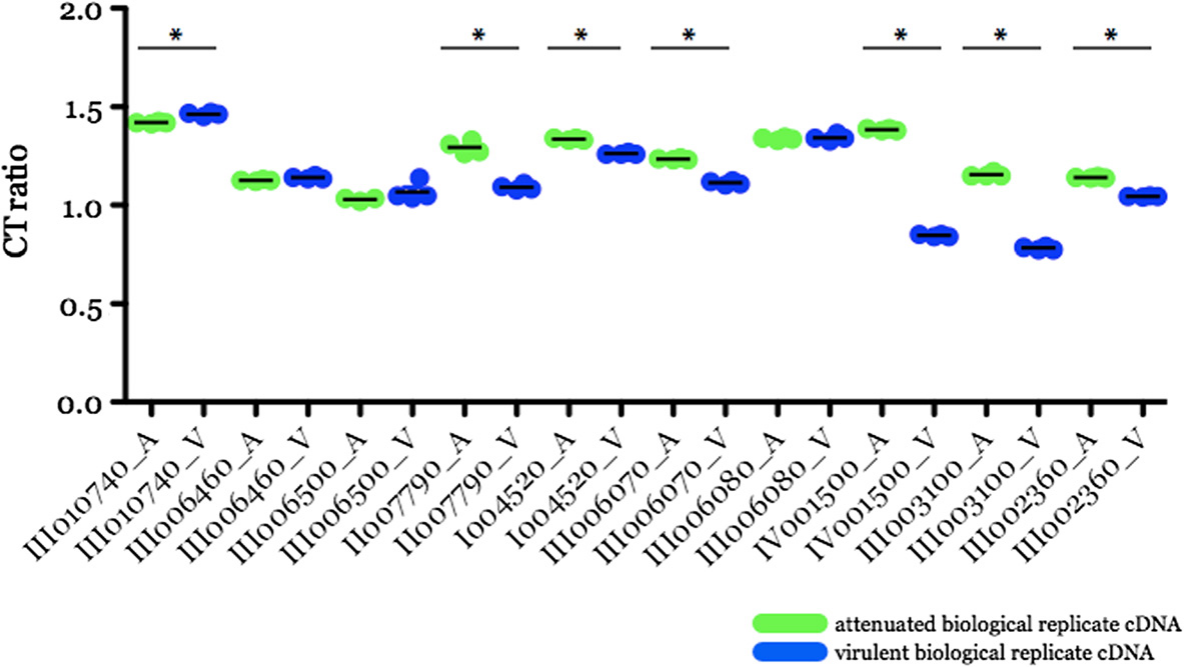
**Validation of ten differentially expressed genes using quantitative PCR on independently generated biological replicate sample sets as templates.** Transcripts were randomly selected for validation and their expression considered significantly regulated (*) if p < .05 using a 1-way ANOVA with Bonferroni post-test analysis (Graphpad Prism v.5.0a). Expression values as cycle thresholds were normalized to those of a house keeping gene, BBOV_III004820, thus, the final data are represented as cycle threshold ratio (CT ratio) where the lower the ratio value, the higher the expression. III 010740, gene encoding for 1-deoxy-D-xylose-5-phosphate reductoisomerase; III006460 and III006500, genes encoding for spherical body protein 2 truncated copies 7 and 9 respectively; II007790, gene encoding for a hypothetical protein; III006070, III001500, I004520 and III003100, genes encoding for variant erythrocyte surface (*ves*) α subunits; III006080 and IV001490, genes encoding for *ves* β subunits and III002360, gene encoding for a putative membrane protein.

Additional differentially regulated genes in T2Bo (V or A) were those encoding for hypothetical proteins, putative membrane proteins, spherical body protein (SBP) 2 truncated copies (11%, n = 4) and 1-deoxy-D-xylose-5-phosphate reductoisomerase (DOXPR) (3%, n = 1) (Figure 2C). Hypothetical and putative membrane proteins are two protein groups whose transcripts were found to be upregulated in the virulent and attenuated strains, though none of the specific protein group members were common between T2Bo_V and _A. *Sbp*2 truncated copies were exclusively upregulated in T2Bo_A by as much as 7 fold (2.8-7.8 folds) as compared to T2Bo_V (Table 2 and Additional file 1: Table S5). These transcripts belong to the *sbp*2 gene family which encodes a large parasite-derived, exported protein that resides on the cytoplasmic side of the infected cell [19,20]. SBP2 is *Babesia*-specific [21,22] and is valuable in diagnostic procedures in suspected *Babesia-*infected cattle [23]. SBP2 and its family members’ functions are currently unknown (Figure 2B) and its upregulation in additional *Babesia* will reinforce its contribution in attenuation acquisition (see RNA-sequencing data below). *Doxpr* transcript was validated to be significantly upregulated in T2Bo_A using a different BR sample pair from those used to generate the microarray data. DOXPR is a critical component in the non-mevalonate pathway (MEP) in the *B. bovis* apicoplast lumen [24]. This pathway is conserved among apicoplast-containing apicomplexans and consists of six major participants in addition to DOXPR. Although *doxpr* was upregulated significantly in T2Bo_A (Figure 3), none of the other MEP participants’ transcripts were upregulated in T2Bo_A. This was unexpected and one could only speculate DOXPR upregulation in T2Bo_A may be involved in a pathway other than MEP in this attenuated strain and its regulation is irrelevant to virulence loss in *Babesia*. Further evidence supporting such implication comes from the RNA-sequencing analysis in L17_A which will be discussed later in the study.

**Table 2:** Spherical body protein 2 gene family members (SBP2) that were significantly upregulated in attenuated (A) T2Bo and L17 strains

**Gene ID**	**Annotation**	T2Bo_A (fold changes)*	L17_A (fold changes)*
BBOV_III005600	SBP2 truncated copy 1	ND	3.3
BBOV_III005830	SBP2 truncated copy 4	7.8	ND
BBOV_III005840	SBP2 truncated copy 5	ND	2.5
BBOV_III006460	SBP2 truncated copy 7	2.8	2.3
BBOV_III006500	SBP2 truncated copy 9	3.9	8.3
BBOV_III006540	SBP2 truncated copy 11	4.6	3.7

*ND*, not detected to be significantly upregulated; *, fold changes is in reference to the respective virulent parental strains.

Among the randomly selected transcripts chosen for microarray data validation in this study (Figure 3), 70% confirmed significant differential regulation while 20% showed differential regulation but were not statistically significant and one transcript showed the opposite trend from the array data (II007790) (p < .05). Including additional validated genes (manuscript in prep) [25], greater than 95% of randomly chosen transcripts were validated suggesting that the microarray assessment of the T2Bo virulent and attenuated transcriptome profiles is representative. One possible explanation to why we were unable to validate a small percentage differentially expressed transcripts may be due to sample pairs used in BR preparation are not clonal, thus may exhibit population variability. Transcripts whose differential expression profiles are not shared between biological replicates may not contribute in the attenuation process.

### RNA-sequencing analysis on *B. bovis* L17 transcriptomes

**Figure 4:**
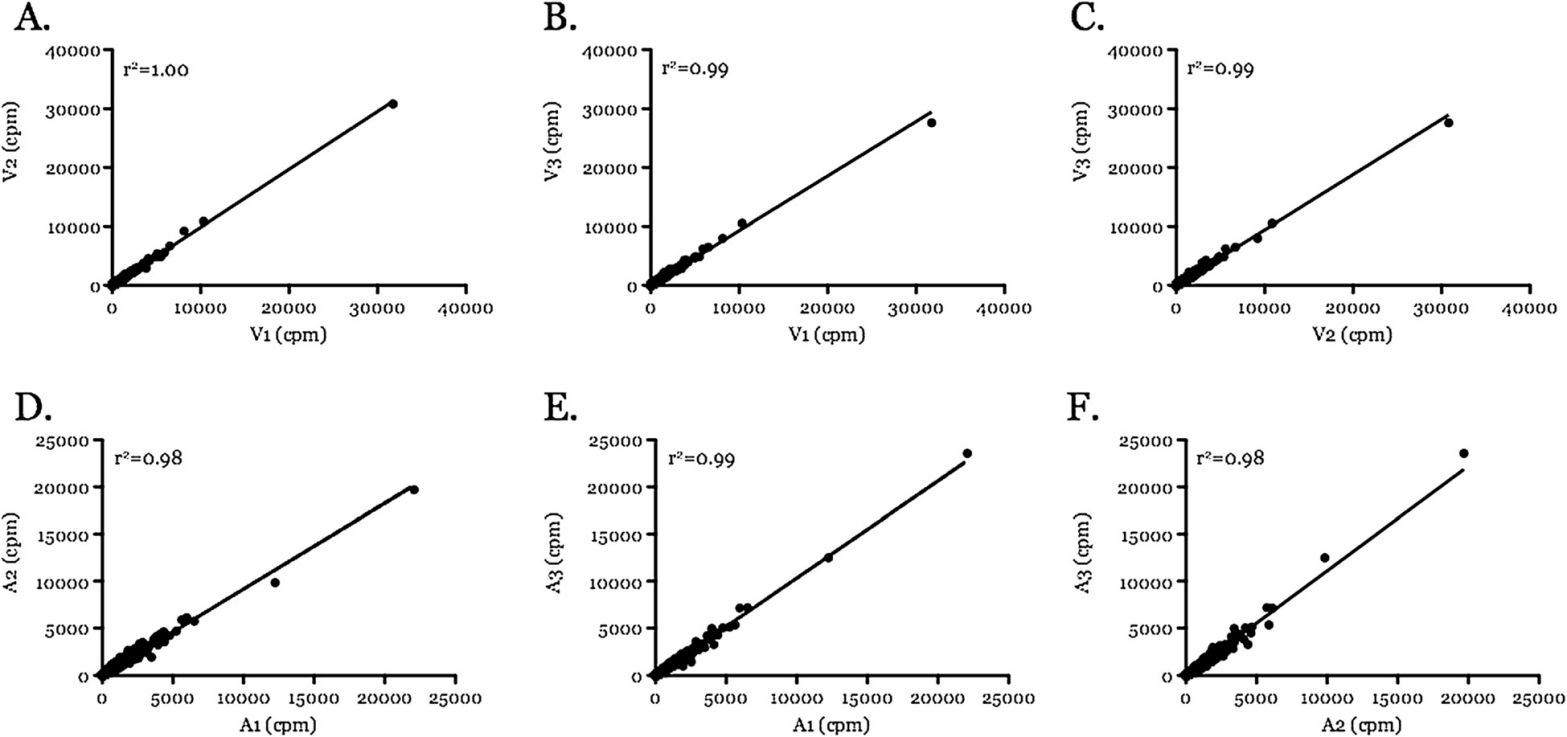
**Correlation plots of signal intensities as counts per million (cpm) were compared between the three L17 biological replicate sample pairs.** Comparisons of each virulent pair **(A-C)** and each attenuated pair **(D-F)** are shown.

**Figure 5:**
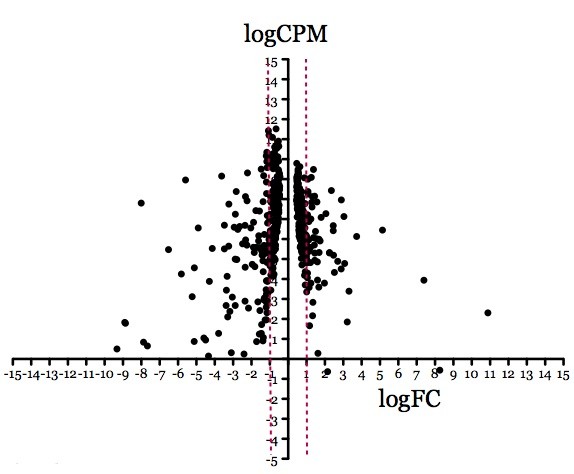
**Plot smear presentation of differentially expressed gene distribution between virulent and attenuated L17 samples.** Genes that are plotted left or right of the dotted red lines are those that were up- or down-regulated by at least two fold. FC, fold changes and CPM, counts per millions.

**Figure 6:**
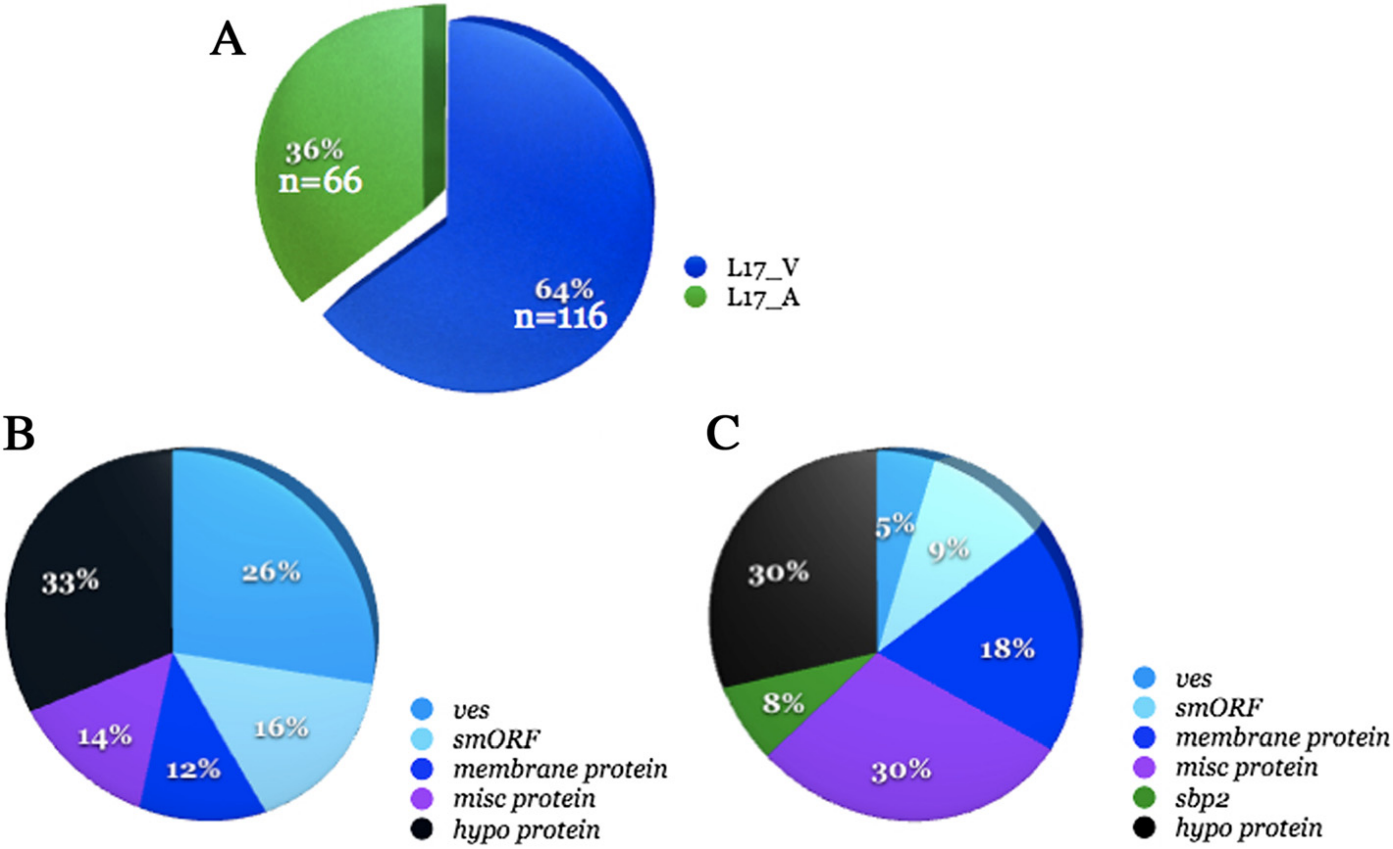
**Global distribution of differentially regulated transcripts in virulent and attenuated L17 *Babesia bovis *strain pair and the breakdown of the putative functions of these transcripts. (A)** A total of 182 (116 + 66) genes were differentially expressed where 64% and 46% were upregulated in the virulent and attenuated strains, respectively. **(B)** Distribution of upregulated genes in the virulent strain and their putative protein functions. **(C)** Distribution of upregulated genes in the attenuated derivative strain and their putative functions. *Ves*, gene encoding for the variant erythrocyte surface antigen (VESA)1; *sbp2*, gene encoding for spherical body protein 2; *smORF*, gene encoding for the small open reading frame protein; misc., miscellaneous genes of various putative functions (as listed in Additional file 1: Table S4-S7).

**Figure 7:**
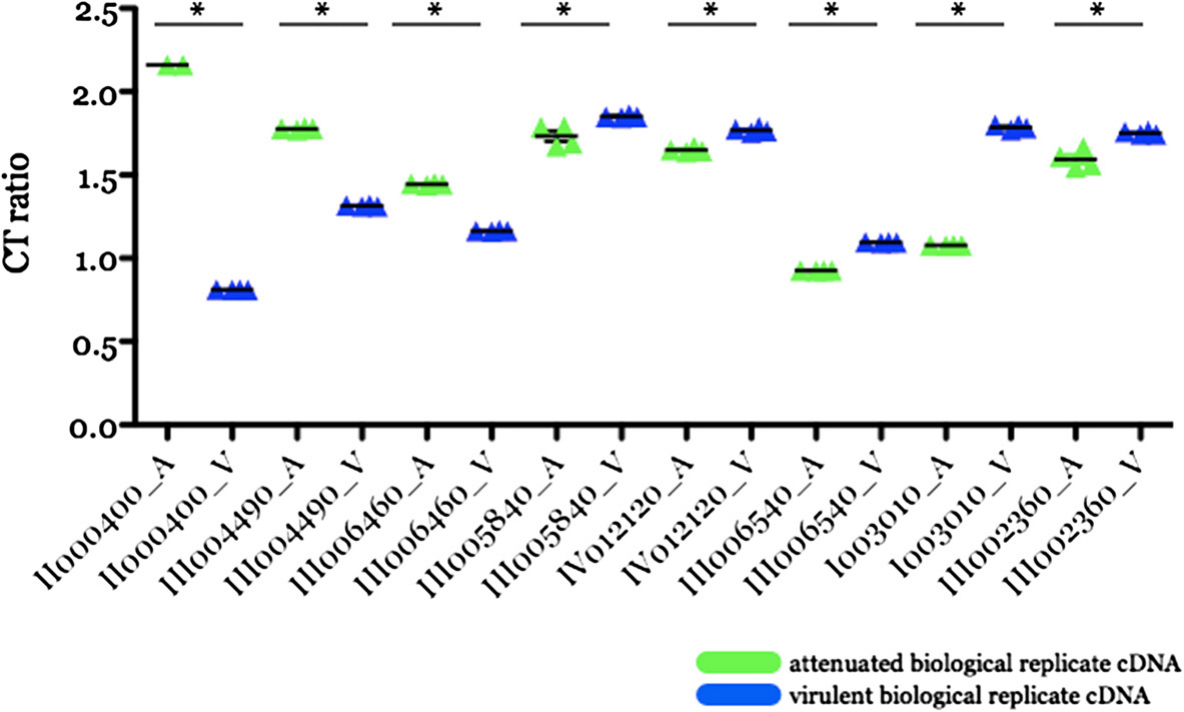
**Validation of RNA-sequencing data with quantitative PCR using independently generated biological replicate sample set.** Only highly regulated transcripts were randomly selected for validation and considered significantly (*) expressed if p < .05 using a 1-way ANOVA with Bonferroni post-test analysis (Graphpad Prism v.5.0a). Expression values as cycle thresholds were normalized to those of a house keeping gene, BBOV_III004820, thus, the final data are represented as cycle threshold ratio (CT ratio) where the lower the ratio value, the higher the expression. I003010, gene encoding for merozoite surface antigen 2a1; III000400, gene encoding for a small open reading frame protein (SmORF); III002360, gene encoding for a membrane protein; III004490, gene encoding for a hypothetical protein; III005840, III006460 and III006540 were genes encoding for spherical body protein 2 truncated copies 5, 7 and 11, respectively; IV012120, gene encoding for a membrane protein.

To investigate if specific differentially expressed transcript profile of one *B. bovis* strain pair (T2B0_V and _A strain) is also observed in a geographically unrelated strain pair, transcriptomic profile of an additional *B. bovis* (L17_V and _A) strain pair was obtained. Three thousand six hundred and thirty-one transcripts were detected using Illumina-based RNA-sequencing technique (96.75% of predicted transcripts in the genome) and compared between L17_V and _A BR samples (n = 3). Correlation plots using counts per million (cpm) of the three BRs ranged between 0.98 and 1.00 and reflect minimal bias in sample preparation (Figure 4). With p value set at 1x10^-5^ as before, four hundred eighty-four transcripts showed significant differentially expression between the L17*_*V and _A samples. These differentially expressed transcripts were further scrutinized by selecting for those whose |logFC| value was greater than 1 (i.e. at least 2 fold difference) (Figure 5), reducing the total number of significant upregulated transcripts in *B. bovis* L17_V and _A to 116 and 66, respectively (Figure 6A). Absolute log fold changes (|logFC|) of these differentially expressed transcripts ranged between 5.2 and 9.3 (Additional file 1: Tables S6-S7). Eight highly differentially regulated genes were chosen for validation using an independently prepared BR L17 sample pair (Figure 7). The number of transcripts chosen for validating the RNA-sequencing results is consistent with previous studies [26] and validation results corroborated with the RNA-sequencing data. Detectable differentially expressed transcripts reported by the array were approximately 3x fewer than those by RNA-sequencing (1.66% vs. 5.01%). Two possibilities may account for the difference. First, RNA-sequencing technology is known to be superior at detecting lower transcript levels than microarray [27]. Second, there are more differentially expressed transcripts in the L17 strain pairs. In this study, it is likely that heightened sensitivity of RNA-sequencing is the reason why more differentially expressed transcripts were detected in the L17 strain pairs. This high sensitivity of RNA-sequencing technology was able to detect approximately 97% of the total predicted transcripts in the *B. bovis* genome. This implies that *B. bovis* asexual stages utilize a large proportion of the predicted protein encoded genes. This, however, does not suggest that these transcripts are important exclusively for the asexual stages, with 3% of the remaining protein encoded transcripts used for other stages. Majority of the detected transcripts in both the virulent and attenuated strain pair (92%) are likely used in stages other than the asexual stages as they were not statistically and significantly different in their regulation between the strain pair.

**Figure 8:**
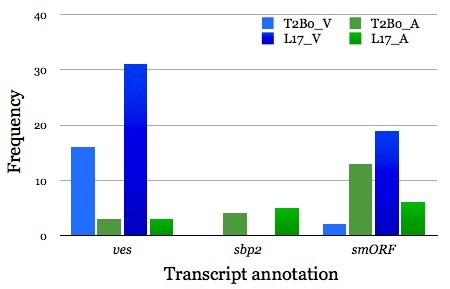
**Comparison of the mean frequencies of major differentially regulated gene families between virulent and attenuated *Babesia bovis *strains*. ****Ves*, gene encoding for the variant erythrocyte surface antigen (VESA)1; *sbp2,* gene encoding for spherical body protein 2 truncated copies and *smORF,* gene encoding for a small open reading frame protein.

Included among the 116 upregulated genes in L17_V were genes encoding for hypothetical proteins (n = 38, 33%), SmORFs (n = 18, 16%), VESA1 (n = 30, 26%), hexose transporters (n = 2, 2%) and other miscellaneous proteins (n = 28, 24%) (Figure 6B). Approximately seven percent (6.9%) of the total upregulated genes in L17_V were also upregulated in T2Bo_V. These were four *ves* (I005140, I004520, IV001500 and IV007980), two *smORF*s (I001370, I001160) and a hypothetical protein-encoding gene (I001350). Two of these upregulated *ves* transcripts in L17_V (α:I004520; β:I004510) form a *ves* αβ “LAT” pair. The remaining upregulated virulent-associated *ves* (α or β) shared between T2Bo and L17 virulent strains were not paired in a divergently oriented manner which are referred to as singletons. Also interestingly is that only a single *ves* divergently oriented αβ pair was detected to be significantly upregulated in L17_V as opposed to three upregulated divergently oriented *ves* αβ pairs in T2Bo_V. As mutually exclusive transcription of this gene family occurs [28], the detection of three *ves* αβ pairs may suggest that the parasites which eventually express these “functional” VESA1 heterodimers represent the dominant members within the heterologous T2Bo virulent population while the detection of a single *ves* αβ pair in L17_V sample may imply that only one dominant parasite which expresses functional VESA1 αβ is present within the L17 virulent population. If divergently oriented *ves* αβ pair’s transcription and subsequent expression are associated with virulence severity, then T2B0_V may be more virulent than L17_V. This datum may also suggest that L17_V contains a less diverse population than T2Bo_V. Unpaired *ves* α/β subunits, regardless of their expression in virulent or attenuated L17 strain, may still contribute to antigenic variability through segmental gene conversion [16]. The higher mean frequency of upregulated *ves* in L17_V than T2Bo_V, regardless of paired or singleton status, is likely to be due to greater sensitivity of the RNA-sequencing technique (Figure 8). Furthermore, there is a possibility that the failure of observing any putative “functional” *ves* αβ transcripts in both attenuated strains could be due to these parasites being sequestered within capillaries as peripheral blood was collected for the preparation of sample pairs [29].

The pattern of differential expression of *smORF* genes in the two strains is unique to each strain. There were fewer upregulated *smORF* genes in T2Bo_V than in L17_V with little overlap between the strains, suggesting that subsequent SmORF proteins may not play a significant role in the virulence phenotype (Figure 8).

Among the genes encoding proteins that were significantly upregulated in L17_A (n = 66), hypothetical (30%, n = 20), putative membrane (18%, n = 12), SmORF (9%, n = 9) and VESA1 (5%, n = 3) remain the major groups (Figure 6C) with 10.6% of the 66 of the total upregulated genes in L17_A also upregulated in T2Bo_A. As similarly found in the T2Bo_A transcriptome profile, the upregulated hypothetical and putative membrane protein encoding genes likely participate in strain-specific activities. Their involvement in virulence loss is still possible but this idea is further dampened by the fact that some of these L17_A-associated upregulated genes were found to be upregulated in T2Bo_V (e.g. I001360, II002820, II007850 and III002360) (Additional file 1: Table S4 and S7).

Genes encoding for SBP2 truncated copies (1, 4, 5, 7, 9 and 11) were upregulated in both attenuated strains (Figure 8). Specifically, *sbp*2 truncated copies 4, 7,9 and 11 were upregulated in T2Bo_A while truncated copies 1, 5, 7 9 and 11 were upregulated in L17_A. This makes *sbp*2 truncated copies 7, 9 and 11 upregulated in both attenuated strains (Table 2, Additional file 1: Table S5 and S7). Multiple sequence alignments of *sbp*2_1, 4, 5, 7, 9 and 11 indicated that they share extremely high sequence homology (e^-200–887^, data not shown) and thus *sbp*2_4 that was upregulated in T2Bo_A could potientially be substituted by *sbp*2_1 and 5 in L17_A. Again, exactly how the truncated copies participate in SBP2’s function and why their upregulation may contribute to virulence loss are unknown. SBP2 has been reported to reside in the cytoplasmic side of the infected erythrocytes. It is possible that this protein family interacts with either parasite- or host-derived proteins, a scenario not foreign to the apicomplexan field (e.g. knob-associated histidine rich protein, KAHRP [30]). Perhaps upregulation of SBP2 family genes and subsequent overexpression of the proteins disrupt the normal matrix of protein-protein interaction, resulting in virulence loss. Experiments investigating into such a phenomenon are currently underway.

In addition to transcripts described above, some miscellaneous transcripts encoding proteins were exclusively upregulated in L17_A by at least 2 fold. These include transcripts encoding for NifU-domain contain protein, HesB-domain containing protein, HAD hydrolase, u6-snRNA associated sm-like Lsm2 protein and oxidoreductase NAD-binding domain containing protein (Additional file 1: Table S7). NifU-domain containing proteins perform basic cellular functions and are among the most highly conserved proteins [31]. HesB-domain containing proteins participate in the formation of metallo-sulfur cluster assembly [32]. HAD hydrolases function in housekeeping detoxification, modulation of sugar-phosphate balance in plants [33] while u6-snRNA associated sm-like Lsm2 proteins have been reported to be involved in RNA processing and may function in a chaperone-like manner in eukaryotes [34]. Lastly, oxidoreductase NAD-binding proteins are known to be enrolled in both aerobic and anaerobic metabolism which include glycolysis, tricarboxylic acid (TCA) cycle, oxidative phosphorylation, and amino acid metabolism [35]. As all these transcripts were solely upregulated in L17_A and not in T2Bo_A, we hypothesize that these transcripts and subsequent proteins, if translated, likely function in processes unrelated to virulence loss.

It was unexpected and disappointing that none of the differentially regulated transcripts with some annotation data involved in immune evasion, infection or reproduction efficiency were detected. This, by no means, suggests that such transcripts were not involved as they may be one of the putative membrane or the hypothetical proteins that were differentially regulated in both strains.

## Conclusions

Two *B. bovis* strain pairs (T2Bo and L17) were used to investigate if virulence loss in protozoans is controlled at the transcriptional level through common strategies. For this study, we utilized a virulent parental phenotype and its attenuated derivative of each strain. We developed and independently validated data generated from an expression microarray for T2Bo and RNA-sequencing for L17, the latter of which yielded three times more differentially regulated genes in the L17 strain pair. We attribute this difference to the heightened sensitivity of RNA-sequencing. Although some differentially expressed transcript families between the two strains were shared, the majority of the specific differentially expressed members were different, suggesting that strain-specific members within common gene families may participate in virulence loss.

We identified upregulation of *ves* αβ pairs in both virulent strains and hypothesize that these “actively and transcriptionally coordinated” *ves* αβ pairs expressed in the virulent strains may contribute to the virulence phenotype. This is consistent with our previous study where we showed that additional *in vivo* serial passage is required to attenuate T2Bo than L17 and more severe clinical pathologies are associated with animals infected with T2Bo_V than those infected with L17_V, all suggest that T2Bo may be more virulent than L17 [14]*.* Specifically, we identified more divergently oriented and actively transcribed αβ pairs in T2Bo_V than L17_V and speculated that T2Bo may be a more heterologous *B. bovis* population with higher virulence than L17. The observation that none of the putative αβ pairs were shared between the two virulent strains could simply mean that different strains utilize different *ves* αβ pairs for the same function.

We also demonstrated that genes encoding for SBP2 truncated copies were significantly upregulated in both attenuated strains, and among these, the specific genes *sbp*2_7, 9 and 11 were upregulated in both. We speculated that *sbp*2 truncated copies 1, 4 and 5, which were not shared, may be used substitutively by the parasite strains in attenuation. Although function of SBP2 is not known, we hypothesized that the upregulation of *sbp2* truncated copies may result in the over expression of the proteins and affect binding partners in the cytoplasm of the infected erythrocytes.

There were additional upregulated transcripts with limited annotational information but they were all strain specific in their expression and may not contribute to the phenotype change due to attenuation.

In summary, our study demonstrates that two *B. bovis* strains underwent identical manipulation/exposure to selection eventually resulting in virulence loss. Despite some similar gene expression profiles shared between the two attenuated derivatives, much of the differential gene regulation is strain specific. Outcome of this study demonstrates the independent adaptation of individual *B. bovis* strains to similar environmental milieu. These data raise further questions inquiring into the composition of the virulent parental strains. Ongoing experiments exploring population dynamics of these virulent *B. bovis* are currently underway.

## Methods

### Parasite strains, *in vitro* cultivation of *B. bovis* and RNA isolation

Two geographical distinct, virulent (V) *B. bovis* (T2Bo and L17 strains) and the generation of their attenuated (A) derivatives were propagated as previously described [14]. Infected blood was collected and frozen stabilates were prepared and stored until use. All animals used for the generation of parasitized erythrocytes were used according to all guideline approved by the Animal Use Committee of the Instituto Nacional de Tecnología Agropecuaria, Argentina. Two and three BR sample pairs for T2Bo and L17 *B. bovis* strains were used in the generation of transcriptome profiles, respectively. These were prepared at different times during a 1-year duration using different animals. For microarray and RNA-sequencing validation, additional BR sample pairs were generated (see below). Quality of the total RNA was evaluated using an Agilent Bioanalyzer.

### Microarray sample preparation

Ten micrograms of total RNA from T2Bo V1-2 and A1-2 were converted to enriched mRNA using Applied Biosystem’s (Ambion) Microb*Express*Tm Kit (AM1905). The manufacturer’s protocol was followed. Enriched mRNA was analyzed by the Agilent 2100 Bioanalyzer. One - 2.5 μg of enriched mRNA from 10 μg of total RNA was obtained. The enriched mRNA (200 ng) was converted to cRNA using Applied Biosystem’s (Ambion) MessageAmp™ II-Bacteria RNA Amplification Kit (AM1790). Amino-allyl-UTP was incorporated into the cRNA during the IVT reaction as described in the manufacturer’s protocol. One hundred μg from 200 ng was obtained. The amine reactive dye Alexa Fluor 555 (Invitrogen A32756) was coupled to cRNA (amino allyl moieties) following the manufacturer’s instructions. Unincorporated dye was removed using RNeasy Mini Columns (Qiagen 74104). The Qiagen quick clean-up protocol was followed. cRNA coupled to fluorescent dye was fragmented using proprietary technology. The expected mean fragment length was 100–125 nucleotides. Fragmented cRNA was considered sufficiently fragmented for use on MYcroarrays if the mean fragment length less than 200 nucleotides.

### Custom 3 × 20 K *B. bovis* arrays

Custom *B. bovis* (Bb) MYcroarrays were manufactured (Mycroarray.com). Briefly, each MYcroarray slide contains three arrays. Each 20 K Bb array had 21,280 potential addresses for features. On each 20 K Bb array, there were 18,490 spots that contain 45mer probes for Bb genes, 2,110 features were “empty” (i.e., they contain no probe), 608 features contain MYcroarray in-house QC probes and 72 features contain positive control probes for assessing hybridization and washing stringency. There were 5 identical replicates, i.e. technical replicates, of each unique Bb probe sequence. A total of 3,698 Bb genes were interrogated on each array, one probe sequence per gene. Designated probes that could cross hybridize due to conserved sequences were noted (Additional file 1: Table S1). A total of 24 genes (23 apicoplast-encoded and 1 nuclear-encoded) were not represented in the array due to the failure of single gene-specific probe design (Additional file 1: Table S2).

### Single color hybridizations

cRNA derived from all samples was coupled to Alexa Fluor 555. Each target (5 μg) was hybridized separately to one array. Hybridization was performed for >18 hrs at 50°C in a proprietary hybridization buffer and subsequent wash steps in MYcorarray’s 1X wash solutionwas carried out twice at 22°C for 3 min, once at 50°C for 3 min with gentle agitation. The arrays were allowed to cool to room temperature in fresh 1X wash solution, transferred to 0.25× wash solution for 30 sec, spun dry in a microarray centrifuge and were scanned in an Axon 4000B Scanner (Molecular Devices) set at 5 micron pixel resolution. The photo multiplier tube (PMT) setting was adjusted to detect the maximum dynamic range of signal (0 to 65,000). This was accomplished by increasing the PMT setting until just a few features (spots) were saturated. The background turned out to be unusually high on two slides. Consequently, the washing process was repeated in exactly the same way with one exception. The 50°C wash was increased to 58°C. Following the second wash procedure, the arrays were scanned at a PMT setting of 380 in the 532 nm (“green”) channel.

### Data analysis

Data were extracted from the scanned images using GenePix Pro Software (version 6.1.0.4). The GAL file (“Load Array List” in GenePix) was positioned over the image. For signal extraction, circular feature indicators (30 μm diameter) were centered over each spot. Median feature pixel intensity was extracted. Flagged features were handled as follows: if a spot was manually flagged as “bad” (a value of −100 in the “flags” field of the .gpr file) no present call was made for this probe and the signal value was replaced with a blank field. Only one array had manually flagged “bad” features (sample A2) and only 303 features were flagged “bad” out of a total of 18,490 (1.6%) Bb features. Since a trimmed mean (see below) was used to estimate the signal for each probe set, the absent value for flagged spots was dropped. Saturated features were handled as follows: To ascertain the full dynamic range of signal intensity, the PMT setting was adjusted until saturation was achieved. In this study, a PMT setting of 380 resulted in some saturated features. There were a total of 211 features with saturation equal to or greater than 10%. This is about 0.2% of the Bb features in the study. The features with ≥10% saturation were handled identically to features that were flagged “bad”. To correct for background, an equation was used: background corrected signal = [Max (signal–background, 0.1) × SF] + C, where C =25 and SF = Scale Factor. In other words, if background subtraction produced a value less than zero, 0.1 replaced the background corrected signal. The background corrected signal was then multiplied by the scaling factor (see normalization below) to adjust for inter-array brightness differences. Finally, the entire signal distribution was shifted to the right by adding constant value (25) to all background corrected and scaled signal values. The shift of the entire signal distribution to the right helped to avoid confoundment of differentiate low signal values from residual noise in the system background noise.

To adjust for differences in dye incorporation, a scale factor was created to equalize signal across all arrays. Only features that met the following criteria were called normalization features (NF) and were used to calculate the scale factor: only Bb features with less than 10% saturated pixels; median signal more than six fold above background and observed in all 6 arrays. There were 12,182 qualifying normalization features. The average signal for the normalization features was calculated for each array while a scale factor (SF) for each array was calculated as follows:

$$\begin{array}{l} \mathrm{SF}=\mathrm{Average}\left(\mathrm{NF}\;\mathrm{median}\;\mathrm{signal}\right)\mathrm{Brightest}\;\mathrm{array}\\ \;/\mathrm{Average}\left(\mathrm{NF}\;\mathrm{median}\;\mathrm{signal}\right)\mathrm{array} \end{array}$$

A scaled F532 median signal for each probe was calculated by multiplying the background adjusted median F532 signal for each feature by the scale factor for each array. Scaling is a minimalistic normalization approach. There are numerous normalization techniques for single color microarray analysis.

A trimmed mean statistic was used to estimate the signal value for each probe set. The median pixel signal for each feature was used after background subtraction and scaling. For each probe set (n = 5) the maximum and minimum value were dropped and the remaining three values were averaged. If there were less than five probe signal values due to either a bad or saturated feature, the trimmed mean was simply calculated on the remaining probes. Using the trimmed mean to estimate signal on an array, is a very simple yet robust approach that helps to mitigate the influence of outlier values and helps normalize intra-array measurements.

An estimation of whether or not a transcript was detected by a probe was calculated. The estimation is a “present” call. The present call is based on the signal value for each gene (probe) relative to the local median background level surrounding each spot. For each probe, if the signal was greater than five times the background signal, then the transcript was considered “present”.

A list of differentially regulated genes was generated. Briefly, each sample group (either A1 vs V1 or A2 vs V2) was compared separately. The filtering criteria were as follows:

•1. For each gene, at least 4 out of 5 probes had to have a present call in one condition (either “A” or “V”).

•2. For the genes that satisfied the present call filter (#1 above), a Student’s *T*-test was performed on the Log2 transformed signal data and only genes with at least p < 0.05 were considered.

•3. Finally, a gene was added to the list if both a fold change ratio (A/V was used arbitrarily) of at least 1.5 combined with a *T*-test p < 0.01.

### RNA-sequencing

Total RNA from L17_V 1–3 and _A1-3 samples were used for the constructions of the libraries as described by the manufacturer (Illumina TruSeq v.2) and carried out at the Fred Hutchison Genomic Center, Seattle. All six samples were multiplexed in a single lane with 50 cycles pair-end runs. The estimated reads per library were 25 million. Following the sequencing process, image analysis and base calling were performed with Illumina RTA v1.13. Reads were aligned to *B. bovis* T2Bo genome (http://www.piroplasmaDB.org) using Tophat v.2.0.4 and Bowtie v.0.12.8. Counts were generated using htseq-count v.0.5.3p9 with the default “union” overlapping mode. Certain genes were removed from consideration if count < 1 per 2 samples. Therefore, the starting # of genes was 3,754 and the post-filtered # of genes was 3,630. Differentially expressed genes pairing the samples by their biological source were identified using edgeR v.2.6.12. Genes were considered significantly and differentially regulated if |log fold change (FC)| ≥ 1 & false detection rate (FDR) < 5%. Additional file 1: Table S3 summaries the htseq counts of the 6 L17 samples.

### Array and RNA- sequencing validation

In order to validate the data obtained by the microarray and RNA-sequencing, qPCR was performed using an independently generated T2Bo or L17 BR sample pair and BBOV_III004820 to normalize the qPCR assay. BBVO_III004820 encodes for a putative topoisomerase II and although its orthologue in *P. falciparum* has been shown to have a four-fold fluctuation between different asexual stages within the erythrocytes [36], its transcript level is indifferent between *B. bovis* V and A via RT-PCR [25]. Primers used for the validation are listed in Additional file 1: Table S8. All primers were confirmed for specificity via cloning and sequencing prior to use (data not shown). Four technical replicates were performed per gene. Cycle thresholds (CT) for a selection of differentially regulated genes were measured. The selection criteria of genes used for the validation process are based on the list of genes that was identified to be significantly differentially expressed in both strains. However, differentially expressed genes selected for validation for the RNA-sequencing data were those that were highly differentially expressed. Significant differentially expressed transcripts with low copy numbers detected by RNA-sequencing likely will not be detectable via qPCR due to its lower sensitivity threshold compared to RNA-sequencing. Upon normalization of the qPCR, relative values of expression were represented as cycle threshold ratios (CT ratio) as previously described [25]. A 1-way ANOVA with Bonferroni post-test analysis was conducted (Graphpad Prism v.5.0a). The list of differentially expressed genes chosen for validation for both assays is in Additional file 1: Table S8.

### Availability of supporting data

All the microarray data have been incorporated into the corresponding genomic data of the *B. bovis* strain (http://www.piroplasmadb.org) while the RNA-sequencing data have been deposited in NCBI’s Gene Expression Omnibus and are accessible through GEO Series accession number GSE51560 (http://www.ncbi.nlm.nih.gov/geo/query/acc.cgi?acc=GSE51560).

## Competing interests

All the authors declared that they have no competing interests.

## Authors’ contributions

MJP, KSS and GMG contributed to data validation and review of the manuscript. IE was responsible for propagating the virulent and attenuated *B. bovis* strain pairs. AOTL was responsible for all experimental design, data analyses and manuscript preparation. MJP is a research associate at Washington State University; KSS is a veterinarian pursuing a PhD in molecular parasitology while completing a microbiology residency at Washington State University. GMG is a Fulbright scholar who is pursuing a PhD in molecular parasitology while IE is a long time collaborator of AOTL, located in Argentina, whose objective is to improve upon the existing bovine babesiosis attenuated vaccine. IE was responsible for providing some of the starting material for this study. AOTL is the principle investigator for the project. All authors read and approved the final manuscript.

## Supplementary Material

Additional file 1: Table S1 *Babesia bovis* genes on the transcriptome array whose probes may cross hybridize. **Table S2.***Babesia bovis* genes that are not represented in the transcriptome array. **Table S3.** Summary of the Illumina based RNA-sequencing on *Babesia bovis* L17 V1-3 and A1-3 samples. **Table S4.** Transcripts that are significantly upregulated (≥2 fold) in *Babesia bovis* T2Bo virulent strain. **Table S5.** Transcripts that are significantly upregulated (≥ 2 fold) in *Babesia bovis* T2Bo attenuated strain. **Table S6.** Top 40 transcripts that are significantly upregulated (≥ 2 fold) in *Babesia bovis* L17 virulent strain. **Table S7.** The top 40 transcripts that are significantly upregulated (≥ 2 fold) in *Babesia bovis* L17 attenuated strain. **Table S8.** Specific primers used for the validation of differentially regulated gene transcripts in T2Bo and L17 *Babesia bovis* strains. Click here for file
