# Supplementary material for: Comparative transcriptome analysis of geographically distinct virulent and attenuated Babesia bovis strains reveals similar gene expression changes through attenuation
Source: BMC Genomics. 2013 Nov 6;14:763. doi: 10.1186/1471-2164-14-763 (PMC3826834; doi:10.1186/1471-2164-14-763)
Supplement: Additional file 1: Table S1 — Babesia bovis genes on the transcriptome array whose probes may cross hybridize. Table S2.Babesia bovis genes that are not represented in the transcriptome array. Table S3. Summary of the Illumina based RNA-sequencing on Babesia bovis L17 V1-3 and A1-3 samples. Table S4. Transcripts that are significantly upregulated (≥2 fold) in Babesia bovis T2Bo virulent strain. Table S5. Transcripts that are significantly upregulated (≥ 2 fold) in Babesia bovis T2Bo attenuated strain. Table S6. Top 40 transcripts that are significantly upregulated (≥ 2 fold) in Babesia bovis L17 virulent strain. Table S7. The top 40 transcripts that are significantly upregulated (≥ 2 fold) in Babesia bovis L17 attenuated strain. Table S8. Specific primers used for the validation of differentially regulated gene transcripts in T2Bo and L17 Babesia bovis strains. [file 1471-2164-14-763-S1.docx]

Table S1. *Babesia bovis* genes on the transcriptome array whose probes may cross hybridize:

Chromosome 1

BBOV_I000010    variant erythrocyte surface antigen-1, beta

BBOV_I000020    variant erythrocyte surface antigen-1, alpha

BBOV_I000030    variant erythrocyte surface antigen-1, beta

BBOV_I001050    hypothetical protein

BBOV_I001130    SmORF

BBOV_I001140    variant erythrocyte surface antigen-1, alpha

BBOV_I001180    SmORF

BBOV_I001190    variant erythrocyte surface antigen-1, beta

BBOV_I001340    variant erythrocyte surface antigen-1, beta

BBOV_I001390    membrane protein, putative

BBOV_I001400    conserved hypothetical protein

BBOV_I001790    conserved hypothetical protein

BBOV_I001810    histone H3, putative

BBOV_I001830    conserved hypothetical protein

BBOV_I001860    histone H3, putative

BBOV_I003000   merozoite surface antigen-2a2 (MSA-2a2)

BBOV_I003010    merozoite surface antigen-2a1 (MSA-2a1)

BBOV_I003830    variant erythrocyte surface antigen-1, alpha

BBOV_I003860    SmORF

BBOV_I003900    variant erythrocyte surface antigen-1, alpha

BBOV_I003910    variant erythrocyte surface antigen-1, alpha

BBOV_I004200    DEAD/DEAH box helicase and helicase conserved

BBOV_I004520    variant erythrocyte surface antigen-1, alpha

BBOV_I005030    conserved hypothetical protein

BBOV_I005040    conserved hypothetical protein

BBOV_I005050    ubiquitin conjugating enzyme, putative

BBOV_I005060    ubiquitin conjugating enzyme, putative

BBOV_I005070    conserved hypothetical protein

BBOV_I005080    conserved hypothetical protein

BBOV_I005110    variant erythrocyte surface antigen-1, alpha

BBOV_I005120    variant erythrocyte surface antigen-1, beta

BBOV_I005140    variant erythrocyte surface antigen-1, alpha

BBOV_I005150    SmORF

BBOV_I005160    variant erythrocyte surface antigen-1, beta

BBOV_I005180    variant erythrocyte surface antigen-1, alpha

BBOV_I005290    variant erythrocyte surface antigen-1, putative

BBOV_I005400    variant erythrocyte surface antigen-1, putative

BBOV_I005410    variant erythrocyte surface antigen-1, putative

BBOV_I005510    variant erythrocyte surface antigen-1, putative

BBOV_I005530    variant erythrocyte surface antigen-1, putative

BBOV_I005650    variant erythrocyte surface antigen-1, putative

BBOV_I005660    variant erythrocyte surface antigen-1, putative

BBOV_I005680    hypothetical protein

BBOV_I029790    hypothetical protein

Chromosome 2

BBOV_II000030   variant erythrocyte surface antigen-1, alpha

BBOV_II000060   SmORF

BBOV_II000080   membrane protein, putative

BBOV_II000110   variant erythrocyte surface antigen-1, alpha

BBOV_II000370   variant erythrocyte surface antigen-1, alpha

BBOV_II000930   hypothetical protein

BBOV_II001390   SmORF

BBOV_II001400   variant erythrocyte surface antigen-1, alpha

BBOV_II001960   hypothetical protein

BBOV_II002260   variant erythrocyte surface antigen-1, alpha

BBOV_II002610   chain A of Ap4a hydrolase protein, putative

BBOV_II002620   u6 snRNA-associated sm-like protein Lsm2

BBOV_II002820   membrane protein, putative

BBOV_II002850   chain A of Ap4a hydrolase protein, putative

BBOV_II002860   u6 snRNA-associated sm-like protein Lsm2

BBOV_II002900   u6 snRNA-associated sm-like protein, putative

BBOV_II002910   chain A of Ap4 hydrolase, putative

BBOV_II004120   variant erythrocyte surface antigen-1, alpha

BBOV_II004160   SmORF

BBOV_II004180   conserved hypothetical protein

BBOV_II004980   hypothetical protein

BBOV_II005950   rhomboid 4

BBOV_II006780   variant erythrocyte surface antigen-1, alpha

BBOV_II006810   SmORF

BBOV_II007350   hypothetical protein

BBOV_II007360   Thioredoxin-like protein 2

BBOV_II007420   hypothetical protein

BBOV_II007430   thioredoxin-like protein 2

BBOV_II007820   SmORF

Chromosome 3

BBOV_III000020  SmORF

BBOV_III000050  SmORF

BBOV_III000060  membrane protein, putative

BBOV_III000070  membrane protein, putative

BBOV_III000090  variant erythrocyte surface antigen-1, beta

BBOV_III000100  variant erythrocyte surface antigen-1, alpha

BBOV_III001140  40S ribosomal protein S17, putative

BBOV_III001170  40S ribosomal protein S17, putative

BBOV_III001180  hypothetical protein

BBOV_III001200  hypothetical protein

BBOV_III001230  hypothetical protein

BBOV_III001240  BBO225AA 22 kDa antigen, fragment

BBOV_III001250  40S ribosomal protein S17, putative

BBOV_III001340  hypothetical protein

BBOV_III001350  40S ribosomal protein S17, putative

BBOV_III002310  variant erythrocyte surface antigen-1, alpha

BBOV_III003640  conserved hypothetical protein

BBOV_III004480  conserved hypothetical protein

BBOV_III004490  hypothetical protein

BBOV_III004500  conserved hypothetical protein

BBOV_III004510  hypothetical protein

BBOV_III005000  WD domain, G-beta repeat containing protein

BBOV_III005010  hypothetical protein

BBOV_III005040  hypothetical protein

BBOV_III005050  WD domain, G-beta repeat containing protein

BBOV_III005580  hypothetical protein

BBOV_III005620  hypothetical protein

BBOV_III005800  hypothetical protein

BBOV_III006050  conserved hypothetical protein

BBOV_III006070  variant erythrocyte surface antigen-1, alpha

BBOV_III006260  60S acidic ribosomal protein P2 (L12EI)

BBOV_III006470  hypothetical protein

BBOV_III006480  Spherical Body Protein 2 truncated copy 8

BBOV_III006490  hypothetical protein

BBOV_III006500  Spherical Body Protein 2 truncated copy 9

BBOV_III006510  hypothetical protein

BBOV_III006520  Spherical Body Protein 2 truncated copy 10

BBOV_III006530  hypothetical protein

BBOV_III006900  HesB-like domain containing protein

BBOV_III006910  conserved hypothetical protein

BBOV_III006930  membrane protein, hypothetical

BBOV_III007100  membrane protein, putative

BBOV_III007130  HesB-like domain containing protein

BBOV_III007490  conserved hypothetical protein

BBOV_III007730  variant erythrocyte surface antigen-1, alpha

BBOV_III007750  hypothetical protein

BBOV_III008070  hypothetical protein

BBOV_III011960  SmORF

BBOV_III011970  variant erythrocyte surface antigen-1, beta

Chromosome 4

BBOV_IV000060   variant erythrocyte surface antigen-1, alpha

BBOV_IV000070   hypothetical protein

BBOV_IV002160   hypothetical protein

BBOV_IV002850   variant erythrocyte surface antigen-1, alpha

BBOV_IV003650   hypothetical protein

BBOV_IV003770   Mtn3/RAG1IP-like protein, putative

BBOV_IV003790   ATP binding family protein

BBOV_IV003800   ATP-dependent Clp protease proteolytic subunit

BBOV_IV003820   Mtn3/RAG1IP-like protein, putative

BBOV_IV003840   ATP binding family protein, putative

BBOV_IV005670   variant erythrocyte surface antigen-1, beta

BBOV_IV005680   variant erythrocyte surface antigen-1, alpha

BBOV_IV006360   senescence-associated protein, putative

BBOV_IV006400   variant erythrocyte surface antigen-1, alpha

BBOV_IV006410   variant erythrocyte surface antigen-1, alpha

BBOV_IV007410   hypothetical protein

BBOV_IV007460   conserved hypothetical protein

BBOV_IV007910   variant erythrocyte surface antigen-1, alpha

BBOV_IV007920   variant erythrocyte surface antigen-1, alpha

BBOV_IV007970   SmORF

BBOV_IV007980   variant erythrocyte surface antigen-1, beta

BBOV_IV007990   variant erythrocyte surface antigen-1, alpha

BBOV_IV008850   hypothetical protein

BBOV_IV008860   hypothetical protein

BBOV_IV009860   rhoptry-associated protein 1 (RAP-1)

BBOV_IV009870   rhoptry-associated protein 1 (RAP-1)

BBOV_IV010620   elongation factor 1-alpha

BBOV_IV010630   elongation factor 1-alpha

BBOV_IV012140   SmORF

BBOV_IV012150   variant erythrocyte surface antigen-1, beta

Table S2. *Babesia bovis* genes that are not represented in the transcriptome array

| Gene ID | Annotation |
| --- | --- |
| BBOV_V000090 | hyp. protein |
| BBOV_V000100 | hyp. protein |
| BBOV_V000140 | hyp. protein |
| BBOV_V000170 | hyp. protein |
| BBOV_V000180 | hyp. protein |
| BBOV_V000190 | hyp. protein |
| BBOV_V000200 | hyp. protein |
| BBOV_V000210 | hyp. protein |
| BBOV_V000280 | hyp. protein |
| BBOV_V000290 | hyp. protein |
| BBOV_V000300 | hyp. protein |
| BBOV_V000310 | hyp. protein |
| BBOV_V000320 | hyp. protein |
| BBOV_V000330 | hyp. protein |
| BBOV_V000350 | hyp. protein |
| BBOV_V000360 | hyp. protein |
| BBOV_V000370 | hyp. protein |
| BBOV_V000380 | hyp. protein |
| BBOV_V000390 | hyp. protein |
| BBOV_V000400 | hyp. protein |
| BBOV_V000420 | hyp. protein |
| BBOV_V000440 | hyp. protein |
| BBOV_V000540 | hyp. protein |
| BBOV_IV002240 | 40s ribosomal protein, s28 |

Table S3. Summary of the Illumina based RNA-sequencing on *Babesia bovis* L17 V1-3 and A1-3 samples

| Gene | L17_A1 | L17_A2 | L17_A3 | L17_V1 | L17_V2 | L17_V3 |
| --- | --- | --- | --- | --- | --- | --- |
| alignment not unique | 8,971,807 | 5,99,0537 | 7,330,267 | 10,166,563 | 8,456,111 | 8,570,119 |
| ambiguous | 700,147 | 581,667 | 661,783 | 696,808 | 594,651 | 573,718 |
| no feature | 1,815,130 | 1,521,953 | 1,504,279 | 1,581,007 | 1,447,098 | 1,445,759 |
| alignments | 28,448,853 | 23,865,077 | 25,595,681 | 27,771,469 | 24,050,972 | 23,798,036 |
| Total | 39,935,937 | 31,959,234 | 35,092,010 | 40,215,847 | 34,548,832 | 34,387,632 |

Table S4. Transcripts that are significantly upregulated (≥2 fold) in *Babesia bovis* T2Bo virulent strain.

| Gene locus tag | P value | Ratio (A/V) (p< .05) | Annotation |
| --- | --- | --- | --- |
| BBOV_II002270 | 6.6E-10 | 0.04 | VESA1α |
| BBOV_III000030 | 10.0E-10 | 0.04 | Membrane protein |
| BBOV_III006070 | 2.7E-12 | 0.1 | VESA1α |
| BBOV_IV001490 | 8.7E-11 | 0.1 | VESA1β |
| BBOV_III003090 | 2.1E-08 | 0.1 | VESA1β |
| BBOV_III006080 | 1.1E-11 | 0.1 | VESA1β |
| BBOV_III003100 | 6.6E-10 | 0.1 | VESA1α |
| BBOV_IV006410 | 2.5E-05 | 0.2 | VESA1α |
| BBOV_I001160 | 2.9E-06 | 0.2 | SmORF |
| BBOV_IV001500 | 1.6E-06 | 0.2 | VESA1α |
| BBOV_I001350 | 5.8E-09 | 0.2 | Hypo. protein |
| BBOV_I001370 | 2.6E-06 | 0.2 | SmORF |
| BBOV_I005120 | 1.6E-08 | 0.2 | VESA1β |
| BBOV_III002360 | 9.9E-10 | 0.2 | Membrane protein |
| BBOV_III007700 | 4.5E-07 | 0.2 | VESA1β |
| BBOV_I005160 | 6.6E-07 | 0.3 | VESA1β |
| BBOV_II007850 | 7.7E-06 | 0.3 | Membrane protein |
| BBOV_I005140 | 5.7E-07 | 0.3 | VESA1α |
| BBOV_I004520 | 5.0E-07 | 0.3 | VESA1α |
| BBOV_II002580 | 1.5E-06 | 0.3 | Membrane protein |
| BBOV_I005510 | 2.9E-08 | 0.3 | VESA1α |
| BBOV_IV007980 | 1.6E-07 | 0.3 | VESA1β |
| BBOV_II000380 | 5.9E-06 | 0.3 | VESA1β |
| BBOV_I001360 | 2.7E-09 | 0.4 | Membrane protein |
| BBOV_II002820 | 1.8E-07 | 0.4 | Membrane protein |

VESA1, variant erythrocyte surface antigen 1; hypo., hypothetical.

Table S5. Transcripts that are significantly upregulated (≥ 2 fold) in *Babesia bovis* T2Bo attenuated strain.

| Gene locus tag | P value | Ratio (A/V) (p< .05) | Annotation |
| --- | --- | --- | --- |
| BBOV_III010730 | 0.002218808 | 2.3 | Hypo. protein |
| BBOV_III011450 | 5.2E-07 | 2.4 | conserved hypo. protein |
| BBOV_II007780 | 6.3E-05 | 2.4 | SmORF |
| BBOV_IV012170 | 8.8E-05 | 2.5 | Hypo. protein |
| BBOV_III010800 | 3.1E-06 | 2.5 | conserved hypo. protein |
| BBOV_I001390 | 1.4E-07 | 2.6 | membrane protein, putative |
| BBOV_II000010 | 4.1E-08 | 2.7 | Hypo. protein |
| BBOV_III006460 | 2.9E-05 | 2.8 | SBP2, truncated copy 7 |
| BBOV_I000040 | 4.4E-07 | 2.8 | VESA1β |
| BBOV_II001380 | 6.6E-08 | 2.8 | SmORF |
| BBOV_III000020 | 4.5E-06 | 2.8 | SmORF |
| BBOV_II002570 | 1.4E-08 | 2.9 | membrane protein |
| BBOV_II007820 | 6.3E-09 | 3.1 | SmORF |
| BBOV_II000400 | 5.2E-07 | 3.1 | SmORF |
| BBOV_II000070 | 5.3E-06 | 3.1 | membrane protein |
| BBOV_III002350 | 8.2E-06 | 3.3 | SmORF |
| BBOV_III011440 | 5.6E-07 | 3.3 | Hypo. protein |
| BBOV_IV000670 | 2.8E-09 | 3.4 | Hypo. protein |
| BBOV_III002320 | 4.0E-06 | 3.5 | VESA1β |
| BBOV_II000670 | 2.2E-08 | 3.6 | Hypo. protein |
| BBOV_IV007970 | 6.8E-10 | 3.8 | SmORF |
| BBOV_III000050 | 0.000249501 | 3.8 | SmORF |
| BBOV_III006500 | 1.2E-09 | 3.9 | SBP2, truncated copy 9 |
| BBOV_III011220 | 2.1E-07 | 4.0 | Hypo. protein |
| BBOV_I005150 | 2.2E-10 | 4.1 | SmORF |
| BBOV_II002280 | 4.2E-08 | 4.4 | SmORF |
| BBOV_III006540 | 5.0E-08 | 4.6 | SBP2 truncated copy 11 |
| BBOV_I005180 | 2.6E-10 | 4.7 | VESA1α |
| BBOV_II007790 | 3.2E-09 | 4.7 | Hypo. protein |
| BBOV_II000060 | 1.6E-08 | 5.1 | SmORF |
| BBOV_IV007960 | 5.6E-10 | 6.2 | SmORF |
| BBOV_III010740 | 0.000161685 | 6.6 | 1-deoxy-D-xylulose 5-phosphate reductoisomerase |
| BBOV_III005830 | 3.4E-09 | 7.8 | SBP2, truncated copy 4 |
| BBOV_III011960 | 1.0E-05 | 11.0 | SmORF |
| BBOV_I003830 | 1.1E-09 | 11.9 | VESA1α |
| BBOV_II004130 | 2.6E-09 | 16.8 | VESA1β |

VESA1, variant erythrocyte surface antigen 1; hypo., hypothetical, SBP2, spherical body protein 2.

Table S6. Top 40 transcripts that are significantly upregulated (≥ 2 fold) in *Babesia bovis* L17 virulent strain.

| Gene locus tag | P value | logFC (≤1) (p<.05) | Annotation |
| --- | --- | --- | --- |
| BBOV_III004490 | 1.3E-34 | -9.3 | hypothetical protein |
| BBOV_IV006430 | 8.3E-63 | -8.9 | SmORF |
| BBOV_IV001040 | 1.8E-61 | -8.9 | hypothetical protein |
| BBOV_II000400 | 0 | -8.0 | SmORF |
| BBOV_IV012120 | 4.0E-40 | -7.9 | membrane protein, putative |
| BBOV_III001180 | 3.6E-36 | -7.7 | hypothetical protein |
| BBOV_IV000350 | 3.3E-173 | -6.5 | VESA1β |
| BBOV_II000080 | 3.6E-125 | -5.8 | membrane protein, putative |
| BBOV_I001160 | 0 | -5.6 | SmORF |
| BBOV_IV001030 | 4.6E-76 | -5.2 | hypothetical protein |
| BBOV_I001310 | 1.0E-30 | -5.1 | membrane protein, putative |
| BBOV_I001170 | 3.6E-92 | -5.1 | SmORF |
| BBOV_IV002830 | 5.9E-124 | -4.9 | VESA1β |
| BBOV_III000690 | 4.7E-32 | -4.6 | SmORF |
| BBOV_II000060 | 4.9E-29 | -4.5 | SmORF |
| BBOV_II004160 | 10.0E-20 | -4.3 | SmORF |
| BBOV_III005970 | 9.2E-80 | -4.3 | membrane protein, putative |
| BBOV_IV012140 | 1.6E-134 | -4.1 | SmORF |
| BBOV_II004150 | 7.6E-29 | -3.8 | SmORF |
| BBOV_IV010150 | 2.0E-187 | -3.6 | hypothetical protein |
| BBOV_IV007930 | 2.2E-138 | -3.5 | SmORF |
| BBOV_II006810 | 4.2E-107 | -3.5 | SmORF |
| BBOV_I001080 | 2.8E-38 | -3.4 | hypothetical protein |
| BBOV_I001390 | 6.3E-42 | -3.4 | membrane protein, putative |
| BBOV_II002300 | 4.5E-41 | -3.3 | VESA1α |
| BBOV_IV000040 | 2.3E-29 | -3.3 | SmORF |
| BBOV_III002700 | 1.3E-86 | -3.2 | hypothetical protein |
| BBOV_IV000360 | 1.5E-165 | -3.2 | membrane protein, putative |
| BBOV_II002590 | 9.4E-31 | -3.2 | membrane protein, putative |
| BBOV_IV011300 | 1.1E-14 | -3.1 | hypothetical protein |
| BBOV_I005650 | 7.8E-13 | -3.0 | VESA1α |
| BBOV_I001350 | 5.8E-93 | -2.9 | hypothetical protein |
| BBOV_I005400 | 5.3E-32 | -2.9 | VESA1 |
| BBOV_III000100 | 6.6E-64 | -2.9 | VESA1α |
| BBOV_III007070 | 8.1E-108 | -2.9 | hypothetical protein |
| BBOV_II002280 | 8.2E-118 | -2.8 | SmORF |
| BBOV_I001370 | 5.1E-59 | -2.8 | SmORF |
| BBOV_I003890 | 1.3E-76 | -2.7 | SmORF |
| BBOV_IV000340 | 9.3E-71 | -2.6 | membrane protein, putative |
| BBOV_I001430 | 2.9E-61 | -2.5 | VESA1α |

SmORF, small open reading frame; VESA1, variant erythrocyte surface antigen 1; FC, fold changes.

Table S7. The top 40 transcripts that are significantly upregulated (≥ 2 fold) in *Babesia bovis* L17 attenuated strain.

| Gene locus tag | P value | logFC (≥1) (p<.05) | Annotation |
| --- | --- | --- | --- |
| BBOV_III010260 | 4.7E-74 | 10.9 | hypothetical protein |
| BBOV_III006500 | 1.3E-20 | 8.3 | SBP2 truncated copy 9 |
| BBOV_III005610 | 1.1E-128 | 7.4 | hypothetical protein |
| BBOV_III010230 | 1.3E-225 | 5.1 | conserved hypothetical protein |
| BBOV_III006540 | 6.1E-128 | 3.7 | SBP2 truncated copy 11 |
| BBOV_III005600 | 3.2E-45 | 3.3 | SBP2 truncated copy 1 |
| BBOV_II002570 | 1.4E-23 | 3.2 | membrane protein, putative |
| BBOV_II007800 | 5.1E-56 | 3.1 | membrane protein, putative |
| BBOV_I003010 | 3.9E-62 | 3.0 | merozoite surface antigen-2a1 (MSA-2a1) |
| BBOV_III002360 | 1.5E-126 | 2.9 | membrane protein, putative |
| BBOV_I001360 | 8.3E-40 | 2.9 | membrane protein, putative |
| BBOV_III006970 | 3.9E-44 | 2.7 | NifU-like domain containing protein |
| BBOV_III006460 | 7.2E-42 | 2.5 | SBP2 truncated copy 7 |
| BBOV_III007130 | 5.2E-39 | 2.5 | HesB-like domain containing protein |
| BBOV_II001390 | 8.4E-60 | 2.5 | SmORF |
| BBOV_III005840 | 1.5E-81 | 2.5 | SBP2 truncated copy 5 |
| BBOV_IV003220 | 2.5E-89 | 2.4 | HAD superfamily hydrolase, putative |
| BBOV_II000020 | 1.2E-46 | 2.3 | membrane protein, putative |
| BBOV_II003660 | 7.9E-49 | 2.3 | conserved hypothetical protein |
| BBOV_II001000 | 1.3E-06 | 2.2 | tRNA-Thr |
| BBOV_I003100 | 1.2E-62 | 2.1 | conserved hypothetical protein |
| BBOV_IV010730 | 2.0E-24 | 2.0 | hypothetical protein |
| BBOV_IV003250 | 2.1E-52 | 1.8 | hypothetical protein |
| BBOV_II002620 | 1.0E-27 | 1.7 | u6 snRNA-associated sm-like protein Lsm2, putative |
| BBOV_IV010720 | 1.3E-25 | 1.7 | hypothetical protein |
| BBOV_II006800 | 4.1E-28 | 1.7 | SmORF |
| BBOV_IV000020 | 3.8E-17 | 1.7 | membrane protein, putative |
| BBOV_III002350 | 3.5E-06 | 1.6 | SmORF |
| BBOV_I002480 | 9.2E-16 | 1.6 | hypothetical protein |
| BBOV_II002610 | 4.5E-20 | 1.6 | chain A of Ap4a hydrolase protein, putative |
| BBOV_III011280 | 5.6E-48 | 1.7 | transporter, major facilitator family protein |
| BBOV_III006190 | 7.7E-29 | 1.5 | conserved hypothetical protein |
| BBOV_I003110 | 2.4E-28 | 1.5 | hypothetical protein |
| BBOV_IV011290 | 3.0E-21 | 1.5 | oxidoreductase NAD-binding domain containing protein |
| BBOV_IV004770 | 9.8E-18 | 1.5 | hypothetical protein |
| BBOV_II000940 | 8.3E-43 | 1.4 | VESA1α |
| BBOV_II002290 | 2.2E-23 | 1.4 | SmORF |
| BBOV_IV007150 | 7.8E-27 | 1.4 | membrane protein, putative |
| BBOV_I003880 | 9.1E-24 | 1.4 | SmORF |
| BBOV_III001210 | 4.5E-10 | 1.3 | membrane protein, putative |

SBP 2, spherical body protein 2; SmORF, small open reading frame; FC, fold changes.

Table S8. Specific primers used for the validation of differentially regulated gene transcripts in T2Bo and L17 *Babesia bovis* strains

| Gene name | Primer sequences (5’ to 3’) | Amplicon size (bp) | Tm (°C) |
| --- | --- | --- | --- |
| BBOV_I004520 | F’: GACTTGCAAATGCGCTTTAGC | 125 | 57.5 |
|  | R’: GCTGATAAATAGCTGTCCTCG |  | 59.8 |
| BBOV_II007790 | F’: TCCAGAGTCACCTAATAGCTCC | 125 | 54.8 |
|  | R’: GCTTGACCACAAACTGCTTG |  | 51.8 |
| BBOV_III002360* | F’: GGTCCTTCTATGGCTCTACTTGTGC | 172 | 59.3 |
|  | R’: CCAGTTCATTGGCGATACGAGG |  | 56.7 |
| BBOV_III003100 | F’: CTACTGAGAGTAATAGTCTAC | 107 | 55.4 |
|  | R’: CGTGCTGCAGTATTTTGGGG |  | 53.8 |
| BBOV_III006070 | F’: GGTCGCCGAGAAAAGGGATGA | 186 | 60.0 |
|  | R’: TGTCTGTTTGGTACCACTGGT |  | 60.6 |
| BBOV_III006080 | F’: GGGGTATTAAATGGTGTAGTA | 189 | 58.1 |
|  | R’: CATAGTGAAGGCCACTAGTGG |  | 58.8 |
| BBOV_III006460_T2BO | F’: GGTTGCCAAGTGGATTGTCG | 189 | 55.1 |
|  | R’: TCGGCGTGAGCAACATTTAC |  | 53.7 |
| BBOV_III006460_L17 | F’: GTTGCCAAGTGGATTGTCGG | 187 | 59.8 |
|  | R’: CGGCGTGAGCAACATTTACA |  | 59.5 |
| BBOV_III006500 | F’: CAACCCATAAAGAGCAAGATCAG | 115 | 53.5 |
|  | R’: TCTGGTCCTTTGCACTGATC |  | 51.8 |
| BBOV_III010740 | F’: CTGATAGGATGGAATCTACGCTC | 128 | 55.3 |
|  | R’: ACAATCCACCCATCTTTCCG |  | 51.8 |
| BBOV_IV001490 | F’: CCATTGATGGCATTCCTGTGT | 117 | 58.5 |
|  | R’: GAATAGTATCCTTGGATTCAAT |  | 57.3 |
| BBOV_IV001500 | F’: ATCATAGACGACATGTTTGAC | 122 | 55.7 |
|  | R’: CATGTATTGTTGGTGGCAGGT |  | 57.9 |
| BBOV_I003010 | F’: CCTGCTGAAACCCAACAAACTC | 197 | 54.8 |
|  | R’: GTCAATCCGCCATAGGTGAATG |  | 54.8 |
| BBOV_II000400 | F’: CTTAGACAACAGATTCCCCCGT | 151 | 54.8 |
|  | R’: TGGTTCATCGCAGTCTTCTGGA |  | 54.8 |
| BBOV_III005840 | F’: GGCTGAAGTTGCTGAAGTGAAGTC | 139 | 57.4 |
|  | R’: CCTACGGTATGCTTTTCCAGGG |  | 56.7 |
| BBOV_III006540 | F’: GAATGGAGAGGATAAGGCGATG | 121 | 54.8 |
|  | R’: TGTAAACGACTTGCCCCTTG |  | 51.8 |
| BBOV_II004490 | F’: GCCTGGAAGTCCCGAAATCA | 139 | 56.3 |
|  | R’: GATTCTCGCAGCGCATTCTG |  | 55.9 |
| BBOV_IV012120 | F’: AGCATACTCCAGGGATGGCA | 173 | 54.3 |
|  | R’: CCGTGTTCTCAGCAGTGTCA |  | 51.7 |
| BBOV_III004820 | F’: GGGATAGGTACAGGATTCAGC | 197 | 54.4 |
|  | R’: TCCGTTTTCATCTAGCCACTG |  | 52.4 |

Green font, gene used for qPCR normalization, *, genes used in the validation of both strains; blue font, genes for the RNA-seq. validation only.
